# Genotyping by Sequencing Highlights a Polygenic Resistance to *Ralstonia pseudosolanacearum* in Eggplant (*Solanum melongena* L.)

**DOI:** 10.3390/ijms19020357

**Published:** 2018-01-25

**Authors:** Sylvia Salgon, Morgane Raynal, Sylvain Lebon, Jean-Michel Baptiste, Marie-Christine Daunay, Jacques Dintinger, Cyril Jourda

**Affiliations:** 1 Centre de Coopération Internationale en Recherche Agronomique pour le Développement (CIRAD), Unité Mixte de Recherche Peuplements Végétaux et Bio-agresseurs en Milieu Tropical (UMR PVBMT), F-97410 Saint-Pierre, France; sylvain.lebon@cirad.fr (S.L.); jean-michel.baptiste@cirad.fr (J.-M.B.); jacques.dintinger@cirad.fr (J.D.); 2 Unité Mixte de Recherche Peuplements Végétaux et Bio-agresseurs en Milieu Tropical (UMR PVBMT), Université de la Réunion, F-97410 Saint-Pierre, France; 3 Association Réunionnaise pour la Modernisation de l’Economie Fruitière Légumière et Horticole (ARMEFLHOR), F-97410 Saint-Pierre, France; 4 NOVA GENETIC, F-49160 Longué-Jumelles, France; morgane.raynal@novagenetic.com; 5 Institut National de la Recherche Agronomique (INRA), Unité de Recherche Génétique et Amélioration des Fruits et Légumes (UR GAFL), F-84143 Montfavet, France; marie-christine.brand-daunay@inra.fr

**Keywords:** *Solanum melongena*, doubled haploid population, bacterial wilt, quantitative trait locus (QTL) mapping, polygenic resistance

## Abstract

Eggplant cultivation is limited by numerous diseases, including the devastating bacterial wilt (BW) caused by the *Ralstonia solanacearum* species complex (RSSC). Within the RSSC, *Ralstonia pseudosolanacearum* (including phylotypes I and III) causes severe damage to all solanaceous crops, including eggplant. Therefore, the creation of cultivars resistant to *R. pseudosolanacearum* strains is a major goal for breeders. An intraspecific eggplant population, segregating for resistance, was created from the cross between the susceptible MM738 and the resistant EG203 lines. The population of 123 doubled haploid lines was challenged with two strains belonging to phylotypes I (PSS4) and III (R3598), which both bypass the published *EBWR9* BW-resistance quantitative trait locus (QTL). Ten and three QTLs of resistance to PSS4 and to R3598, respectively, were detected and mapped. All were strongly influenced by environmental conditions. The most stable QTLs were found on chromosomes 3 and 6. Given their estimated physical position, these newly detected QTLs are putatively syntenic with BW-resistance QTLs in tomato. In particular, the QTLs’ position on chromosome 6 overlaps with that of the major broad-spectrum tomato resistance QTL *Bwr-6*. The present study is a first step towards understanding the complex polygenic system, which underlies the high level of BW resistance of the EG203 line.

## 1. Introduction

Eggplant (*Solanum melongena* L.) is a major vegetable crop in tropical and subtropical regions. It belongs to the very large family of Solanaceae, which includes crops cultivated all over the world, such as tomato (*Solanum lycopersicum*), potato (*Solanum tuberosum*), and pepper (*Capsicum annuum*). Whereas the majority of Solanaceae is endemic to the Americas, eggplant and its wild relatives originated from the old world. DNA sequence data analyses suggest that eggplant originated from Africa and spread throughout the Middle East to Asia [1]. While Asia is the world’s largest producer (47 million tons in 2014; available online: http://www.fao.org/faostat/en/#data/QC), eggplant is also an important crop in Africa and the Mediterranean countries [2]. In addition to its gustative qualities, eggplant is rich in phenolic compounds with antioxidant properties, vitamins, and some minerals [3,4,5,6,7], which means that it is very beneficial for human health. However, its production is limited by pests and diseases, including bacterial wilt (BW), one of the most serious and widespread diseases in the tropics.

BW disease is caused by a soil-borne bacterium belonging to the *Ralstonia solanacearum* species complex (RSSC). It affects more than 250 plant species belonging to 54 plant families [8,9]. The RSSC has been divided into four monophyletic groups called phylotypes, which have each been connected to a potential geographical origin: phylotype I to Asia, phylotypes II (subdivided in IIA and IIB) to the Americas, phylotype III to Africa, and phylotype IV to Indonesia [10]. Each phylotype can be further subdivided in clonal lineages called sequevar [10]. Recent studies proposed a new taxonomic division of the RSSC into three genomic species [11,12]. In this new classification, phylotypes I and III belong to the same species, known as *Ralstonia pseudosolanacearum*, phylotype II remains *R. solanacearum*, but phylotype IV is named *Ralstonia syzygii* subspecies *indonesiensis*. In the absence of a host, the bacterium can survive in moist soils or in water for several years [13]. In the presence of susceptible hosts, the bacterium enters the root, colonizes the xylem vessels, and rapidly spreads to the upper parts of the plant. When the bacterium multiplies, it produces exopolysaccharides that disrupt the water flux, causing the typical wilting symptoms occurring within a few days after infection. The most susceptible plants die, releasing a large quantity of inoculum into the soil. Although the plants colonized by the bacterium very often show little or no symptoms of wilt and although this phenomenon of latent infection or tolerance is important for BW epidemiology [14], the underlying mechanisms have been so far poorly investigated. Given its high soil persistence and its multiplication within asymptomatic hosts, this pathogen is extremely difficult to eradicate from the field.

Management strategies to control BW disease, such as the rotation of host and non-host crops, have produced significant results. However, they appear ineffective when dealing with strains that have a wide host range [15]. The biological control with bacterial phages was also tested, and promising results were obtained in controlled conditions [16,17,18]. However, these results have not been validated in the field. In the last decades, breeding-resistant cultivars proved to be a promising strategy for controlling BW disease, however hampered by the strong interactions between plant resistance and bacterium diversity. Sources of BW resistance are available within Solanaceae genetic diversity and have been the subject of in-depth studies in tomato. The cultivar Hawaii 7996, recognized as the most stable source of resistance to BW disease [19], was used as the resistant parent of the Hawaii7996 × Wva700 interspecific population developed for mapping studies. Several minor and major resistance quantitative trait loci (QTLs) were detected [20,21,22,23,24,25]. The major QTLs *Bwr-6* and *Bwr-12* were detected on chromosomes (chr) 6 and 12, respectively. *Bwr-6* appears to confer resistance to phylotype I and II strains, whereas *Bwr-12* confers resistance only to phylotype I strains. Other minor QTLs were detected on chr 2, 3, 4, 7, 8, 10, and 11, suggesting the presence of a polygenic system in Hawaii 7996. A high level of resistance was also encountered in pepper germplasm. However, latent infection was more frequently observed in this species than in tomato and eggplant [26]. Several resistance QTLs were detected on chr 2, 4, 6, 9, 10, and 11 with both additive and epistatic effects, in a segregating population issued from the resistant PM687 line and a susceptible parent. This suggests that the genetics of resistance in PM687 is complex [27]. In another pepper population derived from the resistant LS2341 line, only one major QTL, *Bw1*, was detected on chr 8 [28,29]. In eggplant, extensive work has been recently conducted on the resistant breeding line AG91-25, whose pedigree supposes the combination of factors of resistance from both *Solanum melongena* and *Solanum aethiopicum* species [30]. The total resistance of AG91-25 over several strains of phylotype I, first identified in climatic chamber conditions [26] and confirmed later in greenhouse, is provided by one major QTL, initially named *ERs1* [31]. *ERs1*, renamed *EBWR9* because of its position on chr 9 [32], specifically confers resistance to a part of phylotype I strains. Two other QTLs, *EBWR2* and *EBWR5*, detected on chr 2 and 5, were found to encode partial resistance to strains of phylotypes I and phylotypes IIA and III, respectively [32]. *EBWR2*, the single QTL detected with the highly virulent strains PSS4 and TO10 (phylotype I), delays disease progression but it is clearly unable to provide a sufficient resistance level. Thus, researching other sources of resistance is necessary for breeding cultivars resistant to strains which bypass the resistance conferred by the AG91-25 QTLs.

In this perspective, the *Solanum melongena* Indian line EG203 is of particular interest. Indeed, this line, challenged with a core collection of 12 RSSC strains belonging to three phylotypes [26], was demonstrated to be highly resistant to 7 of them, moderately resistant to 4, and moderately susceptible to 1. EG203 was also found resistant to a set of phylotype I strains from Ivory Coast [33]. Consequently, the objectives of this study were to:

(i) Genotype a population of doubled haploid lines (DH) obtained from the hybrid [EG203 (encoded E4) × susceptible line MM 738 (encoded E8)] and construct a dense genetic map anchored on the tomato genome.
(ii) Challenge this population with *R. pseudosolanacearum* (=phylotypes I and III) because it is the most harmful RSSC type in major eggplant production areas [33,34,35,36,37], and map EG203 resistance QTLs.
(iii) Compare the position of EG203 resistance QTLs with those reported in other eggplant populations as well as in other solanaceous crops, thanks to the synteny of their genomes.

The two strains used in this study (PSS4 and R3598) belong to phylotype I sequevar 15 (I-15) and phylotype III sequevar 29 (III-29), respectively.

## 2. Results

### 2.1. A Dense New Anchored Genetic Map for Eggplant

**Figure 1:**
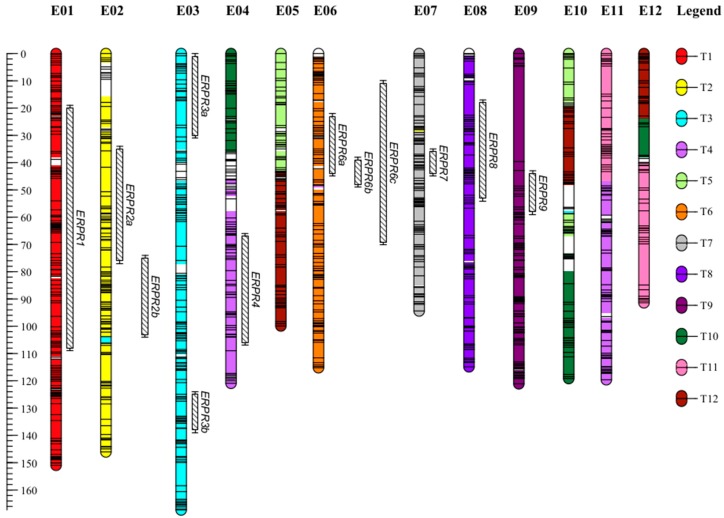
Genetic map of EG203 × MM738 doubled haploid population anchored on eggplant and tomato chromosomes. The figure shows the 12 linkage groups (LG)–chromosomes from E01 to E12 and their corresponding tomato chromosomes individualized by a color code (key on the right of the figure). The markers’ positions are symbolized by horizontal lines on the LG bars; the markers’ names were not included to facilitate map legibility. The list and positions of all markers can be found in Table S3. The detected quantitative trait loci (QTLs) and their confidence intervals are represented on the right of their respective chromosomes by black hatched bars, and their names are on the right of each QTL bar.

The sequencing of genotyping by sequencing (GBS) libraries generated a total of 3.4 × 10^9^ 150 bp reads. The fastQC analysis conducted on cleaned reads revealed the absence of remaining Illumina adapters. After the cleaning and the demultiplexing step, 56% of the initial reads were discarded and 20 × 10^6^ and 23 × 10^6^ reads were attributed to E8 and E4 parents, respectively. The population of (E4 × E8) DH lines yielded a mean of 9.9 × 10^6^ reads, with a minimum of 1.8 × 10^6^ and a maximum of 19.6 × 10^6^ (for more details, see Table S1). A total of 476,479 loci were constructed with the de novo pipeline. Among them, 24,237 were polymorphic (5%). After applying the different filters, 6336 single-nucleotide polymorphisms (SNPs) were identified. A total of 1370 SNPs genotyped for 123 DH lines were analyzed using JoinMap. The markers formed 13 linkage groups (LG) at independence logarithm of odds (LOD) from 4 to 6. Two LG were merged because they had reciprocal Strongest Cross Link (SCL) values at LOD > 3. The resulting 12 LG had a total of 1170 SNPs. Among the 1170 loci sequences bearing SNPs, 1154 (98.6%) were aligned on eggplant’s contigs, 505 (43.2%) were aligned on the potato genome, 456 (39%) on the tomato genome, and 325 (28%) on the pepper genome. Thanks to these anchor markers, the 12 LG could be tracked back to the 12 eggplant chromosomes (E01 to E12), as defined by Hirakawa et al. [38] and anchored on the tomato genome (Figure 1). The LG ranged from 91.39 to 167.34 centimorgans (cM) and harbored 53 to 141 SNPs. The map had a high density with an average of 1 SNP every 1.25 cM. Chromosomes E08, E09, E11, and E12 had large gaps (>10 cM). E09 had the largest gap with a distance of 34.90 cM between two adjacent SNPs. The genetic map had 7.44% of distorted SNPs (*p* < 0.05), with E10 having the highest proportion of distorted SNPs (29%). Among the 33,873 eggplant contigs belonging to 56 eggplant–tomato synteny blocks (sb) [38], 863 contigs covering 55 sb were anchored in our map. The map was estimated to cover 86% of the genome. Statistics of the map are provided in Table S2, and genotypic data are detailed in Table S3.

### 2.2. Segregation of Resistance in the Doubled Haploid Population 

**Figure 2:**
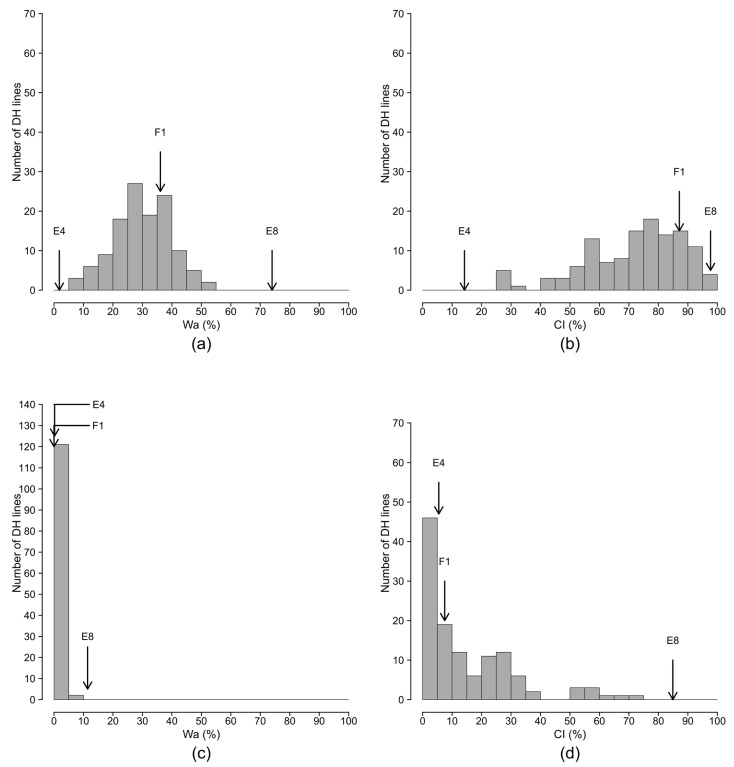
Frequency distributions of the percentage of wilted plants (W_a_) and colonization index (CI) variables in the EG203 × MM738 doubled haploid (DH) population inoculated with strains PSS4 (Reunion assay) and R3598 (Cameroon assay). The data were combined across seasons. The frequency distribution of the W_a_ and CI variables are presented, respectively, in (**a**,**b**) for strain PSS4 and (**c**,**d**) for strain R3598; the arrows indicate the means of susceptible parent E8 (MM738), resistant parent E4 (EG203), and their F_1_ (E4 × E8). The W_a_ variable was obtained from the best linear unbiased predictor (BLUP) model for strain PSS4.

The maximum score (SCO_max_) and percentage of wilted plants (W_max_) variables were highly correlated in individual seasons and across the seasons in both the Reunion Island and Cameroon trials (Pearson correlation coefficient from 0.93 to 0.99, Tables S4 and S5). In the same way, the areas under disease progress curve of both score (SCO_a_) and percentage of wilted plants (W_a_) variables were highly correlated (Pearson correlation coefficient from 0.98 to 0.99). On the basis of these high correlations, we present only the analyses for SCO_max_, W_a_, and CI (colonization index) variables. The frequency distributions for W_a_ and CI variables are indicated in Figure S1 (individual seasons) and Figure 2 (data combined across seasons). The distributions for the SCO_max_ were very similar to the distributions of W_a_ and are not presented. 

With PSS4 tested in Reunion, the frequency distributions of best linear unbiased predictors (BLUPs) of W_a_ and CI were continuous and approximately fitted to a Gaussian curve (Figure 2a,b). The BLUPs of W_a_ was skewed toward the resistant parent (E4) with the F_1_ position intermediate between E8 and E4 (Figure 2a), whereas CI was skewed toward the susceptible parent (E8) with F_1_ positioned close to the E8 parent (Figure 2b). According to the phenotypic groups (P_g_) generated by fuzzy analysis (Table 1), the E8 parent was highly susceptible (P_g_ = 5) in both seasons, whereas E4 was highly resistant in season 1 (P_g_ = 1) and moderately resistant in season 2 (P_g_ = 2) (Table 1, Figure S1a–d). The backcross with the susceptible parent E8 (BC_1_E8) was highly susceptible (P_g_ = 5), whereas the backcross with the resistant parent E4 (BC_1_E4) was moderately resistant (P_g_ = 2) (Table 1). The F_1_, F_2_, and DH progenies were moderately susceptible (P_g_ = 4) in both seasons. Among the controls, E9 was the most resistant, whereas E10 was the most susceptible. The susceptibility of E6 (AG91-25) to PSS4 strain, observed by Lebeau et al. [31], was confirmed in both seasons. Anova revealed a highly significant genotypic (G) effect (*p* < 0.001) for the three variables in both individual and combined seasons (Table 2). The repetition (R) effect was not significant for SCO_max_ and W_a_, but was significant for CI (*p* < 0.01 and *p* < 0.001 in individual and combined seasons, respectively). The season (S) effect was highly significant for all three variables (*p* < 0.001). The interaction between genotype and season effects (G × S) was significant (*p* < 0.05 for CI and *p* < 0.01 for SCO_max_ and BLUPs of W_a_). The heritability (h^2^) ranged from 0.34 to 0.70 for season 1, from 0.36 to 0.58 for season 2, and from 0.50 to 0.71 for combined seasons (Table 2).

With R3598 tested in Cameroon, the frequency distributions did not fit a Gaussian curve (Figure 2c,d). The BLUPs of W_a_ was highly skewed toward resistance (Figure 2c) with parents E8 and E4 positioned very close to each other. CI was also skewed toward resistance, but the parents were located near the extremes (Figure 2d). E8 was tolerant (P_g_ = 3.2, latent infection) in season 1 (i.e., plants stayed asymptomatic despite a high quantity of bacteria detected inside the xylem vessels) and moderately susceptible (P_g_ = 4) in season 2, whereas E4 was highly resistant (P_g_ = 1) in both seasons. F_1_ was also highly resistant in both seasons (P_g_ = 1), suggesting a dominant inheritance of resistance. F_2_ was moderately resistant in season 1 (P_g_ = 2) and tolerant in season 2 (P_g_ = 3.2). BC_1_E8 was tolerant (P_g_ = 3.2), whereas BC_1_E4 was highly resistant (P_g_ = 1) in both seasons. In the controls, E9 was the most resistant (P_g_ = 1 for both seasons) and E10 was the most susceptible, although its phenotypic group was tolerant (P_g_ = 3.2). As SCO_max_ and W_a_ could not be approximated by a Gaussian model, Anova was only conducted on the binary CI variable (0 for noncolonized and 1 for colonized). The genotype effect for CI was highly significant in both individual and combined seasons (*p* < 0.001, Table 2). In combined seasons, Genotype, Season, Repetition, and G × S effects were all significant with h^2^ ranging from 0.39 to 0.56 (Table 2).

### 2.3. A Polygenic System Involved in the Resistance to PSS4 and R3598 Strains 

Given the different patterns of distribution in the Reunion (strain PSS4) and Cameroon (R3598) trials, different analytical models were used to detect the QTLs involved in the resistance to each strain. 

For PSS4 data, SCO_max_, BLUP W_a_, and CI variables were first analyzed using the simple interval mapping method (SIM) with a normal model. According to the season and the variable, six QTLs of resistance to PSS4 were identified (Table S6). These QTLs explained from 9.3 to 19.2% of the phenotypic variance (R^2^). Significant epistatic interactions were found between the detected QTLs. Models including the QTLs’ additive and epistatic effects explained up to 35.7% of the total phenotypic variance. The detected QTLs were season-dependent. These results suggest that there is a polygenic system of resistance that is highly influenced by environmental conditions. The existence of a polygenic system was confirmed by using a multiple QTL mapping (stepwise analysis), for which a total of 10 QTLs were detected (Table 3). 

In season 1, a single QTL (*ERPR3a*) was detected with SCO_max_ and BLUP of W_a_, whereas seven QTLs, including *ERPR3a* (*ERPR1*, *ERPR2a*, *ERPR3b*, *ERPR4*, *ERPR6a*, and *ERPR7*), detected with CI, explained from 4.8% (*ERPR1*) to 17% (*ERPR3b*) of the phenotypic variance (Table 3). A significant epistatic interaction between *ERPR4* and *ERPR6a* was detected and accounted for 4% of the phenotypic variance. The models that included all detected QTLs explained 13.2 (SCO_max_), 11.9 (W_a_), and 65.4% (CI) of the total phenotypic variance. 

In the second season, four QTLs (named *ERPR2b*, *ERPR4*, *ERPR6b*, and *ERPR8*) were detected with both SCO_max_ and BLUP of W_a_, whereas a single QTL (*ERPR1*) was detected with CI (Table 3). These QTLs explained from 8% (*ERPR4*) to 25.3% (*ERPR6b*) of the phenotypic variance. The models explained 45.7, 40.4, and 10.9% of the total phenotypic variance, respectively, for SCO_max_, W_a_, and CI. 

In combined seasons, five QTLs (*ERPR2b*, *ERPR3a*, *ERPR4*, *ERPR6b*, and *ERPR8*), four QTLs (*ERPR2b*, *ERPR4*, *ERPR6b*, and *ERPR8*), and six QTLs (*ERPR1*, *ERPR2b*, *ERPR3a*, *ERPR3b*, *ERPR4*, and *ERPR6a*) were detected with the SCO_max_, W_a_, and CI, respectively (Table 3, Figure 1). The QTLs detected explained from 6.4% (*ERPR3a*) to 21.7% (*ERPR6b*) of the total phenotypic variance. The models that included all the QTLs detected were able to explain 54.0%, 44.9%, and 56.9% of the total variance. 

For data generated with the R3598 strain, the SCO_max_, W_a_, and CI variables were also analyzed, first using SIM with a non-parametric model (Table S7). One QTL (*ERPR3a*) was detected for SCO_max_, W_a_, and CI in the first season, and only for CI in combined seasons. Another QTL (*ERPR9*) was only detected in the second season for SCO_max_ and CI. 

The variables were then coded as binary, and the resulting SCO_b_, CI_10b_, and CI_b_ variables were used for the stepwise analysis (Table 3). In the first season, one QTL (*ERPR3a*) was detected for SCO_b_ and CI_10b_, which explained 11.4 and 29.7% of the total variance, respectively. Two QTLs (*ERPR3a*, *ERPR6c*) were detected for CI_b_, explaining 21.4 and 12.0% of phenotypic variance individually and 31.3% of the total phenotypic variance (Table 3). *ERPR3a* and *ERPR6c* were also detected for the combined season for the CI_b_ variable, which explained 12.6 and 10.3% of the phenotypic variance, respectively. *ERPR3a*, the only QTL detected for CI_10b_, explained 25.8% of the phenotypic variance for the combined seasons. Only one QTL was detected in season 2. This QTL, *ERPR9*, was specifically detected for CI_10b_ and explained 15.0% of the phenotypic variance. 

Taken together, these results suggest that there are several QTLs which confer resistance to PSS4 with minor to medium effects, and only a few QTLs which confer resistance to R3598. Resistance to both strains is provided by a common QTL (*ERPR3a*), as well as by three QTLs (*ERPR6a*, *b*, *c*), which are almost colocalized (Table 3, Figure 1). The results indicate the strong influence of the seasons on the expression of resistance. The negative additive effects found for all the QTLs detected (Table 3) indicate that resistance originates exclusively from E4 (EG203) alleles. As expected, no QTLs of resistance originate from E8, the highly susceptible MM738 parent.

### 2.4. Epistatic Effects Influence the Resistance to PSS4 Strain

In order to identify putative digenic interactions which are not detected by the stepwise analysis, interaction plots (effectplots) between each pair of detected QTLs were constructed for SCO_max_ for combined seasons. The graphs in Figure S2 suggest digenic interactions between *ERPR2b*/*ERPR6b* (S2c), *ERPR2b*/*ERPR8* (S2d), *ERPR3a/ERPR6b* (S2f), *ERPR3a*/*ERPR8* (S2g), *ERPR4*/*ERPR6b* (S2h), and *ERPR4*/*ERPR8* (S2i). The least significant difference (LSD) test confirmed the *ERPR2b*/*ERPR6b*, *ERPR2b/ERPR8*, *ERPR3a*/*ERPR6b*, *ERPR3a/ERPR8*, and *ERPR4/ERPR8* interactions (Table 4). In the case of the *ERPR2b*/*ERPR6b* and *ERPR3a*/*ERPR6b* pairs of loci, only DH lines homozygous for the resistant alleles at both loci (BB/BB) had a significantly reduced disease score (*p* < 0.05). There was no significant difference between the other three groups of DH lines (genotypes AA/AA, AA/BB, or BB/AA). In the *ERPR2b*/*ERPR8*, *ERPR3a*/*ERPR8*, and *ERPR4*/*ERPR8* pairs of loci, DH lines with genotypes AA/BB, BB/AA, BB/BB had similar disease scores, which were significantly lower (*p* < 0.05) than those of the lines homozygous for the susceptible alleles (AA/AA). Thus, both “more than additive” and “less than additive” types of interactions appeared between pairs of QTLs in the DH population:

- “More than additive” interactions (BB alleles of the resistant parent had a greater effect in the QTL duo combination, than individually) were observed for *ERPR2b*/*ERPR6b* and *ERPR3a*/*ERPR6b* pairs of loci.
- “Less than additive” interactions (BB alleles had a lower effect in combination than individually) were observed for *ERPR2b*/*ERPR8*, *ERPR3a*/*ERPR8*, and *ERPR4*/*ERPR8* pairs of loci.

## 3. Discussion

### 3.1. SNPs from GBS Made It Possible to Construct a Dense New Intraspecific Genetic Map

Although GBS produced several thousand polymorphic SNPs, only 1170 SNPs were included in the final map. We removed a large number of SNPs because they clustered on the genetic map and were, thus, noninformative. DH populations originate from a single meiosis (recombinant inbred line populations arise from several meioses), thereby promoting the clustering of markers because of insufficient recombination events. Despite these limitations, our map displayed a high marker coverage (1 marker every 1.25 cM), covering 55 of the 56 eggplant–tomato synteny blocks (sb) and 86% of the genome, according to the Fishman et al. method [39]. However, as reported in other studies, genome coverage can also be estimated by simply dividing the LG length by the estimated genome length [40,41]. Using this calculation, our map should cover 98% of the eggplant genome. We were able to align 40% of the SNP sequences on the tomato genome, 43% on the potato genome, and 28% on the pepper genome. The marker order in our map was mostly concordant with the physical order of eggplant contigs. 

### 3.2. Several QTLs Are Involved in the Resistance to R. pseudosolanacearum PSS4 and R3598 Strains and Are Possibly Syntenic with Proven Tomato BW-Resistance QTLs

A total of 12 resistance QTLs were detected on eggplant chromosomes 1, 2, 3, 4, 6, 7, 8, and 9 (Figure 1). Three QTLs (*ERPR6a*, *ERPR6b*, and *ERPR6c*) were detected on chr 6. The first two were detected with PSS4 (I-15) strain, whereas the third one was only detected with R3598 (III-29). We distinguished them on the basis of the difference in their confidence intervals for SCO_max_, W_a_, and CI. However, differentiating the three QTLs is debatable because *ERPR6c* confidence interval overlapped with those of both *ERPR6a* and *ERPR6b* (Figure 1). Thus, it may be better to consider these three QTLs as the same *ERPR6* QTL, which provides resistance to both PSS4 and R3598 strains. If we consider a unique QTL on chr 6, our results point out the presence of: (i) seven QTLs specific to PSS4 strain (*ERPR1*, *ERPR2a*, *ERPR2b*, *ERPR3b*, *ERPR4*, *ERPR7*, and *ERPR8*), (ii) one QTL specific to R3598 (*ERPR9*, although only detected in season 2), and (iii) two QTLs encoding resistance to both strains (*ERPR3a* and *ERPR6*). Because of the limited size of our population and the different environmental conditions between Reunion Island and Cameroon assays, we cannot exclude that there are more QTLs conferring resistance to both PSS4 and R3598 strains. The apparent specificity of our QTLs to one strain could be caused by a lack of power in our QTL analysis and the presence of genotype–environment interactions. In future work, it could be interesting to phenotype the population with strain R3598 in Reunion Island and strain PSS4 in Cameroon. These assays would help to verify the strain specificity of our detected QTLs.

The low frequency of symptoms observed on the DH population inoculated with strain R3598, might be due to a poor multiplication of this strain at the high temperatures observed in our greenhouse assays (up to 48 °C), given it was collected in altitude (690 m) in Cameroon and thereby might be adapted to cooler temperatures. The weakness of infestation in greenhouse is supported by the relatively low W_a_ values observed for the susceptible parent E8 and the controls E6 and E10 (W_a_ = 11.4%, 0.2% and 3.6% for E8, E6, and E10, respectively) (Table 1). In comparison, in a preliminary assay conducted in a naturally infested field with the strain R3598 in Cameroon, we observed W_a_ values of 39.4, 12.7, and 51.4% for the E8, E6, and E10 lines, respectively (data not shown). Hence, additional tests are needed, either with higher inoculum pressure or in naturally infested fields, in order to ascertain the QTLs providing resistance to R3598. If confirmed as broad-spectrum QTLs, *ERPR3a* and *ERPR6* would be of particular interest for breeding cultivars resistant to *R. pseudosolanacearum* strains which bypassed *EBWR9* [31,32].

Thanks to the anchored markers on the eggplant and tomato genomes, the physical positions of QTLs were estimated and compared with already published eggplant and tomato BW-resistance QTLs. Interestingly, the confidence intervals (37.6–43.9 megabases (Mb) and 43.5–53.1 Mb) of *ERPR2a* and *ERPR2b*, specific to PSS4 strain, overlapped with the position of the recently identified eggplant *EBWR2* [32] QTL (38.3–46.9 Mb) (Table 5). Further, *ERPR3b*, also specific to strain PSS4 and positioned between 124 and 139 cM, corresponded to a narrow physical area of 2.6 Mb (between 65.2 and 67.8 Mb) which overlapped with the physical interval of *Bwr-3*, a tomato QTL detected in the line Hawaii 7996 (Table 5). *ERPR4*, detected on chr 4 between 66 and 107 cM, with a corresponding physical interval located between 59.8 and 65.4 Mb, could also match the position of Hawaii 7996 *bwr-4* QTL, at the bottom of chr 4, but *bwr-4* position was too vague to ascertain the syntenic relationship between both QTLs. The same situation was found for *ERPR6*, conferring resistance to both PSS4 and R3598 strains, possibly syntenic to tomato broad-spectrum *Bwr-6* QTL (Table 5), also of imprecise location. *Bwr-6* was first detected as one broad QTL peak on chr 6 in tomato lines Hawaii7996 and L285 [23,24,42], and a later temporal QTL analysis suggested the presence of two QTLs acting at different stages of the infection [22]. Carmeille et al. [21] considered as one and the same these colocalizing QTLs on chr 6 and kept the name *Bwr-6* [21]. Wang et al. [20] fine-mapping differed according to the phenotype dataset, but these authors concluded that environmental conditions were responsible of *Bwr-6* position shift [20]. Interestingly, the position of *ERPR6* on the short arm of chr 6 is, as for *Bwr-6*, also highly influenced by the phenotypic dataset. The clustering of resistance genes at a single locus has been reported for several plant species [43,44,45]. These clusters can span large chromosomal segments and are implicated in resistance to different races of the same pathogen as well as to different pathogens [46]. In the common bean, resistance to anthracnose was previously described as the result of broad-spectrum single major genes conferring resistance to several races [47]. However, later studies suggested that these single broad-spectrum genes could be a cluster of race-specific resistance genes [48,49]. Therefore, eggplant *ERPR6* and tomato *Bwr-6* loci could be broad-spectrum QTLs as well as a cluster of strain-specific genes. Further inoculations of our DH population with PSS4 and R3598 strains should be repeated in different locations or conditions, in order to conclude about the presence of one or several resistance QTLs on chr 6.

Lastly, the confidence intervals of the remaining QTLs, *ERPR1* (0.1–92.1 Mb), *ERPR7* (3.7–58.0 Mb), *ERPR8* (0.6–58.2 Mb), and *ERPR9* (4.3–33.5 Mb) are too large to allow any reliable comparison of their position with the published ones. 

### 3.3. Breeding Cultivars Resistant to R. pseudosolanacearum Strains Which Bypassed EBWR9 

For decades, breeding BW-resistant cultivars has been limited because of the absence of identified resistance QTLs or genes and the lack of knowledge of the pathogen’s genetic diversity and of its interaction with plant resistance. Since the publication of the concept of bacteria phylotypes [10], the idea of specific or nonspecific relationships between phylotypes or strains and resistance QTLs has emerged. Interestingly, both major specific genetic factors (*Bwr-12* in tomato, *EBWR9* and *RE-bw* in eggplant) and broad-spectrum QTLs were proven to be involved in BW resistance. Thus, both quantitative and qualitative resistances exist in *Solanaceae* and can be used to create cultivars with potentially broad-spectrum and durable resistance. The SNPs produced by GBS will allow the development of molecular markers for cumulating specific and nonspecific resistance factors within breeding lines. Sequences harboring the SNPs can be converted into breeder-friendly markers with Kompetitive Allele Specific PCR (KASP) [50] or high-resolution melting PCR (HRM) [51] and used in marker-assisted selection (MAS). The number of QTLs involved, as well as the large confidence intervals, will however complicate the process of simultaneously introducing several QTLs into a single cultivar. Furthermore, effect plots analysis carried out between pairs of loci has revealed putative epistatic interactions between QTLs (Figure S2). This analysis supports the presence of digenic interactions. *ERPR8* seemed to be involved in “less than additive” interactions with *ERPR2b*, *ERPR3a* and *ERPR4* (Table 4), a case of interaction occurring when several loci are implicated in the same function [52]. On the contrary, *ERPR6b* seemed to be involved in a “more than additive” interaction with *ERPR2b* and *ERPR3a.* This synergic interaction generally occurs when several genes encode for enzymes involved in the same molecular pathway [52]. Hence, digenic interactions should be taken into account when pyramiding QTLs because they may have an adverse effect on the success of detection, introgression, and the characterization of genes underlying the QTLs [53].

Our results also suggest that *ERPR8* can be introgressed without *ERPR2b*, *ERPR3a*, and *ERPR4* and still significantly increase the level of resistance. On the other hand, *ERPR6b* should be introgressed with both *ERPR2b* and *ERPR3a* in order to benefit from their “more than additive” effects. In short, the breeding effort should focus on the introgression of *ERPR2b*, *ERPR3a*, and *ERPR6b* QTLs, which have a synergistic interaction. The DH lines, which are homozygous for the resistant allele at these three loci, displayed a W_a_ mean of 16.4% and a CI mean of 47.7%, (seasons combined). In comparison, the respective values of their counterparts, which are homozygous for the susceptible alleles, were higher (35.6% and 84.1%; data not shown). Thus, the three QTLs combined are expected to reduce disease progression and colonization of the xylem vessels by a factor of 2. Their introgression, together with *EBWR9*, *EBWR2*, and *EBWR14* from the line AG91-25 [32], should be considered seriously for the creation of an outstanding broad-spectrum resistant cultivar. As *ERPR2b* and *EBWR2* colocalize (Table 5), their introgression will be simplified with the use of the same markers. Another important gene of resistance, *RE-bw*, was found in eggplant E-31 inbred line [54], but its spectrum of efficiency has not been explored yet. This gene was cloned and conferred total resistance to a RSSC strain expressing the PopP2 type-III effector. Therefore, further investigations are necessary before the use of this major gene in combination with our BW-resistance QTLs, which have been characterized towards well-characterized and classified strains.

The limited size of our DH population and the simultaneous QTL detection and QTL effects estimation may have generated statistical artifacts. These statistical artifacts, firstly described by Beavis [55,56], were demonstrated through simulation and experimental studies. The authors of these studies showed that QTL mapping and QTL effects estimation in a same population leads to an overestimation of the QTL effects [57,58,59,60]. The phenotypic variances and QTL effects overestimations were most severe for populations of limited sizes [61,62,63]. Because of the limited size of our own population (123 DH lines), the phenotypic variances and QTL effects are likely upward biased and this is supported by the presence of QTL models explaining a total phenotypic variance superior to the heritability of the trait. This bias can also happen when the model is overfitted. Therefore, before introgressing QTLs into commercial cultivars using a MAS program, our QTLs must be validated within the same mapping population and between different mapping populations [59,60]. Meanwhile, given the complex genetic architecture of EG203 resistance, its use as a resistant rootstock could be the best effective strategy as it is already demonstrated for tomato production in both tropical lowlands and highlands [64,65].

### 3.4. Looking for a Complementary Source of Resistance in Eggplant Germplasm

*R. pseudosolanacearum* (phylotype I and III) includes strains that are particularly aggressive and virulent on solanaceous crops [26,33]. Phylotype I has the highest evolutionary potential [66]. This suggests that the resistance conferred by a single major resistance genetic factor, such as *EBWR9*, which is effective against some strains of this phylotype [31,32] could be broken down by the pathogen within a more or less short period. Therefore, in order to breed durable and broad-spectrum resistant cultivars, major and complementary QTLs should be identified and then pyramided into a same genotype. In this way, the lines E3 (MM152) and E9 (S56B), which exhibited high levels of resistance [26], are complementary sources of resistance to both strains PSS4 and R3598 (Table 1). INRA, UR1052, has recently developed populations of DH lines from the crosses MM152 × MM738 and S56B × MM738 that are conserved by the Genetic Resources Center for Vegetable Species (CRB-Leg). These will be used for a wider and in-depth dissection of BW resistance factors in future. The eggplant species complex, which includes the three crops, *S. melongena*, *S. macrocarpon*, and *S. aethiopicum* and their wild relatives (*such as S. linnaeanum*, *S. torvum*, *S. incanum*), represents a tremendous reservoir of genetic diversity [67]. This diversity should also be explored in the future, with a view to finding additional BW-resistance QTLs and genes. 

## 4. Materials and Methods 

### 4.1. Plant Material and DNA Extraction

A DH population of 132 lines was developed using a culture of F_1_ plants from the cross EG203 × MM738. The parent MM738 (E8) is an *S. melongena* line that is highly susceptible to BW. This line was supplied by INRA (Avignon, France) and was also used to produce the recombinant inbred line population MM738 (E8) × AG91-25 (E6) [31]. The parent EG203 (E4), or Surya, is an *S. melongena* line resistant to a broad range of RSSC strains [26], in particular to strains pathogenic to AG91-25. It was supplied by AVRDC (Taiwan). Genomic DNA was extracted from the dried leaves using the Qiagen DNeasy plant mini kit (Qiagen, Courtaboeuf, France), according to the manufacturer’s instructions. Genomic DNA was quantified using a Qubit 2.0 fluorometer (Thermo Fisher Scientific, Illkirch, France), and DNA concentrations were normalized to 50 ng/µL. The quality and homogeneity of the DNA were checked using agarose gel electrophoresis.

### 4.2. Library Construction and Sequencing

The DNA samples of the 132 DH lines and the two parent lines were used for genotyping by sequencing (GBS), using three 96-plex plates. Each plate contained a DNA duplicate of 44 DH lines and both parents. The DNAs were digested with the restriction enzyme ApeKI and ligated with barcode adapters according to the protocol developed by Elshire et al. [68]. The adapters comprised a set of one common adapter and 96 unique barcode sequences detailed in Table S1. Each library was sequenced on three lanes of Illumina HiSeq3000 sequencer with DNA-seq single-read protocol, using the GeT-PlaGe platform (Toulouse, France).

### 4.3. Sequence Analysis and Identification of SNPs

Low-quality reads, reads with uncalled bases, and reads with Illumina adapter sequences were removed using the cutadapt software [69]. The remaining reads were checked using the FastQC tool (Available online: https://www.bioinformatics.babraham.ac.uk/projects/fastqc/) [70]. Then, each sequence was attributed to its corresponding individual using unique barcode adapters with the barcode splitter tool (Available online: https://sourceforge.net/projects/gbsbarcode/). Demultiplexed sequences were trimmed to 140 bp to normalize the length between individuals. The single-nucleotide polymorphisms (SNPs) were identified using the de novo pipeline in the STACKS software version 1.28 (Available online: http://catchenlab.life.illinois.edu/stacks/) [71]. A list of the SNPs present in at least 40% of individuals was generated using sequence alignments with the population module. SNPs with an allele frequency below 5% in the DH lines were discarded because these very rare variants probably resulting from genotyping errors. SNPs with a heterozygosity rate >5% in the DH lines were also discarded because they probably resulted from erroneous loci. As DH lines are supposed to be fixed (in the homozygous state), all lines with a heterozygosity rate >10% were discarded. Heterozygous positions in the population were coded as missing data and then imputed using the missForest package [72] in the R software version 3.3.1 (Available online: https://www.r-project.org/).

### 4.4. Genetic Map Construction

A genetic map was constructed using filtered SNPs with the JoinMap^®^ 4.1 software (Wageningen, Netherlands) [73]. The similarity between each pair of markers was computed. For each pair with similarity =1, one marker was removed (unaligned markers or markers with the highest rate of missing data) to speed up the analysis. The remaining SNPs were grouped with the independence Logarithm of odds (LOD) score option. Two linkage groups were merged if they contained markers with a reciprocal strongest cross-link (SCL) value at LOD > 3. Probable misgrouped markers (with a recombination frequency >0.6) were discarded. Markers were then ordered with the regression mapping algorithm using the default parameter in JoinMap (Available online: https://www.kyazma.nl/index.php/JoinMap/) and the Kosambi function to compute genetic distances. Markers with mean chi-square estimation >2 and genotype probabilities (−Log 10 (P)) > 0.10 may contain genotyping errors and do not fit the map very well. Thus, these markers were discarded, and the linkage groups were reordered. The estimated genome length (*L*) of each linkage group was computed according to the method 4 described by Chakravarti et al. [74]: L=(m+1)/(m−1), where *m* is the number of markers per linkage group. Then, the map coverage was estimated as ${c=1-e}^{-2dn/L}$, where *d* is the average marker interval, *n* is the number of markers, and *L* the estimated genome length [39]. The loci sequences bearing SNPs were aligned on the eggplant (*Solanum melongena*) genome (SME_r2.5.1; [38]), the tomato (*Solanum lycopersicum*) genome (SL2.50; [75]), and the potato (*Solanum tuberosum*) genome (PGSC_DM_v4.03; [76]) using the Blastn program in the NCBI’s BLAST+ software (version 2.2.28, available online: ftp://ftp.ncbi.nlm.nih.gov/blast/executables/blast+/2.2.28/) [77], with a cut-off value of 1 × 10^−20^ for the eggplant genome and 1 × 10^−15^ for the tomato and potato genomes. A map was drawn using MapChart 2.2 (Available online: https://www.wur.nl/en/show/Mapchart.htm) [78].

### 4.5. Bacterial Strains and Inoculum Preparation

Two strains, PSS4 and RUN3598 (R3598), belonging to the *Ralstonia pseudosolanacearum* species were used in this study. The PSS4 strain is a phylotype I sequevar 15 (I-15) from Taiwan, which bypasses major QTL *EBWR9* [32], and the R3598 strain is a phylotype III sequevar 29 (III-29) from Cameroon, which was found to be virulent on the AG91-25 line in the field [79]. The strains were grown on 2,3,5-triphenyl tetrazolium chloride medium [80] for 24 h at 28 °C for the inoculum preparation. The actively growing bacteria were harvested and diluted in Tris buffer (Trizma 0.01 M pH 7.2: Sigma, St. Louis, IL, USA) to obtain a final concentration of 1 × 10^8^ colony-forming unit (CFU) per mL, which was measured using the optical density (OD) value at a wavelength of 600 nm by spectrophotometry (OD_600nm_ = 0.1).

### 4.6. Greenhouse Trials and Phenotyping

Phenotyping trials were conducted in two greenhouses at the experimental station of the Centre de Coopération Internationale en Recherche Agronomique pour le Développement (CIRAD) in Saint-Pierre, Reunion Island (140 m elevation, 21° S, 55.3° E) and in one greenhouse split in two blocks at the experimental station run by the SEMAGRI Company in Yaoundé, Cameroon (690 m elevation, 3.7° N, 11.6° E). Trials in Reunion Island were conducted during two seasons in the two greenhouses (representing four repetitions). Trials in Cameroon were conducted in two seasons in the two blocks, representing four repetitions. Table 6 shows the details for each assay, including the strain used for the inoculation and the environmental conditions.

For both locations, each repetition contained 100 plants of the E8 (MM738) and E4 (EG203) parental lines, 20 plants of the F_1_(E4 × E8), 100 plants of F_2_(E4 × E8), 100 plants of each backcross (BC_1_E8 and BC_1_E4), and 5 plants of each 123 DH lines. Twenty plants of each eggplant control accession, MM152 (E3), AG91-25 (E6), S56B (E9), and MM136 (E10) were used to evaluate the strains’ aggressiveness and virulence. Three-week-old plantlets were transplanted in the greenhouse according to a randomized complete block design. After two weeks of acclimatization in the greenhouse, the plants’ roots were scarified with a knife just before inoculation, in order to ensure a satisfactory infestation. Each plant was inoculated with 100–200 mL at 10^6^ CFU per mL through the drip irrigation system in Reunion Island. In Cameroon, the inoculation was conducted by manually adding in each plantlet pot 30 mL of inoculum at 4 × 10^6^ CFU per mL. The disease symptoms were evaluated twice a week starting 5 and 8 days after inoculation in Reunion Island and Cameroon, respectively. The trials lasted 33 days in Reunion Island and 40 days in Cameroon. A disease scoring scale from 0 to 4 was used, as defined in Lebeau et al. [31]. A plant was considered to have wilted if the score was above 0. At the end of each trial, the presence of latent infection (colonization) was tested, as described in Lebeau et al. [31].

### 4.7. Statistical Analysis of Traits

All the descriptive statistics were carried out using the R software (version 3.3.1, available online: https://www.r-project.org/) [81]. The score (SCO), which is the mean of the disease scoring rate (scale from 0 to 4), the percentage of wilted plants (W), and the percentage of colonized plants (CI) were computed for each of the lines and progenies. The lines and progenies were attributed to one of the six phenotypic groups (P_g_) using the fuzzy analysis clustering method, as defined in Lebeau et al. [26]: 1 = highly resistant, 2 = moderately resistant, 3.1 = partially resistant, 3.2 = latent infection, 4 = moderately susceptible, 5 = highly susceptible. The area under the disease progress curve (AUDPC) was computed for the score (SCO_a_) and wilting percentage (W_a_) as follows: $\sum_{i=1}^{n-1}\frac{X_{i}{+X}_{i+1}}{2}\times \left(t_{i+1}{-t}_{i}\right)\times \frac{1}{t_{n}{-t}_{1}}$, where *X_i_* is the SCO or W value at the *i*th date, *t_i_* is the time at the *i*th observation, and *n* the total number of observations. The Pearson coefficient of correlation was computed between maximal SCO (SCO_max_), maximal W (W_max_), CI, SCO_a_, and W_a_ variables. The analysis of variance (anova) was conducted on the SCO_max_, the W_a_, and the CI variables using the lme4 package [82]. The CI was analyzed as a binomial variable (plants were scored as colonized or non-colonized) with a generalized linear model (glm), whereas the SCO_max_ and W_a_ were analyzed with a linear model (lm). Anova was conducted on variables computed for individual seasons, as well as across the seasons (comb). The following models were used: $y_{ij}=µ+G_{i}{+R}_{j}{+\varepsilon }_{ij}$ and $y_{\mathrm{ijk}}=\;µ+G_{i}{+R}_{j}{+S}_{k}{+\;(G\times S)}_{\mathrm{ik}}{+\varepsilon }_{\mathrm{ijk}}$ for individual seasons and across the seasons, respectively. y is the observed value for the given variable, *µ* is the mean value, *G*, *S*, *R*, *G* × *S* are the genotype, season, repetition nested in season, interaction between genotype and season effects, respectively, and ε is the random error. The chi-square test was used to test the significance of the effects. Broad-sense heritabilities (*h*^2^) and their Bayesian confidence interval were estimated with the MCMCglmm package [83] with a number of Markov chain Monte Carlo (MCMC) iteration fixed to 500,000. The SCO_max_, SCO_a_, and W_a_ were analyzed with a distribution assumed as “Gaussian”, and the W_max_ and CI were analyzed with a distribution assumed as “ordinal”. For individual seasons and across the seasons, respectively, *h*^2^ was calculated as follows: $h^{2}=\frac{V_{G}}{V_{G}+\;\frac{V_{e}}{j}}$ and $h^{2}=\frac{V_{G}}{V_{G}+\;\frac{V_{G\times S}}{k}+\;\frac{V_{e}}{j\times k}}$ where *V_G_* is the genotypic variance, *V_G×S_* is the variance of genotype season interaction, *V_e_* is the environment variance, *j* is the number of repetitions in a season (*j* = 2), and *k* is the number of seasons (*k* = 2). For the analysis of binary variables, an additional source of variance of 1 due to the probit link was added to the formulae: $h^{2}=\frac{V_{G}}{V_{G}+\;\frac{V_{e}}{j}+1}$ and $h^{2}=\frac{V_{G}}{V_{G}+\;\frac{V_{G\times S}}{k}+\;\frac{V_{e}}{j\times k}+1}.$ The best linear unbiased predictors (BLUPs) were extracted from the same models (described above), with *G* and *G* × *S* as the random effect, and S and R as the fixed effect. BLUPs were extracted only for the SCO_a_ and W_a_ because they were the only variables with continued and Gaussian distribution.

### 4.8. QTL Analysis

QTL analysis was performed using the R/qtl package version 1.39 (Available online: http://www.rqtl.org/; [84]). The SCO_max_, the BLUP of W_a_, and the CI traits were analyzed with simple interval mapping (SIM, [85]) with a 1 cM step. Variables with near-normal distribution were analyzed with the normal model and the Haley & Knott (hk) regression [86]. Variables with skewed distribution were analyzed with the non-parametric model implemented in the “scanone” function of R/qtl. The LOD threshold for declaring significant QTLs was determined using a permutation test with 1000 repetitions for a significance level of *p* ≤ 0.05 [87]. The variables were analyzed using multiple QTL mapping (MQM) with the automated stepwise model selection implemented in the “stepwiseqtl” function [88]. Then, 2D permutation tests of 1000 repetitions were run using the hk regression with the “scantwo” function. The function gives the penalties to be used in the stepwise analysis. Models with a maximum of 8 QTLs with both additive and epistatic effects were tested in the MQM analysis. The variables with near-Gaussian distribution were analyzed with a normal model. The variables with severe deviations from normality were analyzed with a binary model. In this way, the SCO_max_ was coded as “0” when the value was =0 and “1” when the value was >0 and renamed SCO_b_. The W_a_ was also coded in the same way as SCO_max_. However, as the two binary variables were 100% correlated, only one of them (SCO_b_) was used for the analysis. The CI were coded as “0” when the value was =0 and “1” elsewhere and renamed CI_b_, or coded as “0” when the value was <10 and “1” elsewhere and renamed CI_10b_. For both SIM and MQM analysis, the positions of the detected QTLs were refined using the “refineqtl” function, and variance components were estimated with the “fitqtl” function. For each QTL, the 95% Bayesian credible intervals were calculated using the “bayesint” function. The digenic epistatic interactions between QTL pairs were examined by constructing plots of means with the “effectplot” function of R/qtl. The digenic interactions were further analyzed by comparing the mean variables for “AA” (E8) and “BB” (E4) genotypes at each pair of QTL (represented by its closest marker), using Fisher’s least-significant-difference test (LSD) [89]. The QTLs detected were named the ERPR “linkage group” (ERPR for Eggplant *Ralstonia Pseudosolanacearum* Resistance). When several QTLs were detected on the same LG at different positions, a letter was added to their QTL name for identification purposes. 

## Figures and Tables

**Table 1:** Mean and standard deviation (in parenthesis) for parental lines, progenies of the cross EG203 (E4) × MM738 (E8), doubled haploid (DH) and control lines, challenged with PSS4 and R3598 *R. pseudosolanacearum* strains.

Strain	Season	Var ^a^	Parents		Progenies of E8 × E4					Control Lines			
			E8	E4	F_1_	F_2_	BC_1_E8	BC_1_E4	DH	E3	E6	E9	E10
PSS4	1	SCO_max_	3.7 (0.1)	0.0 (0.0)	2.2 (0.4)	2.2 (0.1)	3.6 (0.1)	0.6 (0.0)	1.8 (0.1)	0.3 (0.0)	2.2 (1.0)	0.0 (0.0)	3.7 (0.1)
		W_a_	71.5 (2.1)	0.4 (0.4)	35.2 (4.8)	38.1 (2.0)	63.7 (1.2)	12.7 (1.8)	34.3 (1.0)	7.4 (0.5)	39.3 (18.9)	0.0 (0.0)	67.6 (1.7)
		CI	95.3 (2.7)	4.1 (2.0)	87.1 (7.1)	75.6 (1.9)	94.0 (3.0)	38.2 (7.3)	73.7 (1.2)	15.4 (0.4)	90.0 (0.0)	17.2 (12.2)	92.5 (2.5)
		P_g_	5	1	4	4	5	2	4	2	4	1	5
	2	SCO_max_	4.0 (0.0)	0.2 (0.1)	2.3 (0.4)	2.6 (0.2)	3.5 (0.1)	0.5 (0.2)	2.7 (0.1)	0.6 (0.4)	3.4 (0.4)	0.2 (0.2)	3.3 (0.3)
		W_a_	76.6 (0.7)	3.3 (1.2)	37.0 (8.6)	46.0 (5.3)	62.2 (2.9)	8.5 (2.0)	41.4 (1.4)	13.6 (9.6)	60.3 (12.5)	2.4 (2.4)	57.8 (9.2)
		CI	100.0 (0.0)	24.4 (8.2)	87.1 (2.9)	76.4 (6.3)	93.4 (3.6)	29.9 (9.7)	82.0 (1.4)	49.1 (14.1)	95.0 (5.0)	37.2 (7.2)	89.4 (0.6)
		P_g_	5	2	4	4	5	2	4	2	5	2	5
	Comb	SCO_max_	3.8 (0.1)	0.1 (0.1)	2.2 (0.2)	2.4 (0.2)	3.5 (0.1)	0.6 (0.1)	2.2 (0.1)	0.5 (0.2)	2.8 (0.6)	0.1 (0.1)	3.5 (0.2)
		W_a_	74.0 (1.7)	1.8 (1.0)	36.1 (4.1)	42.0 (3.2)	62.9 (1.4)	10.6 (1.6)	34.3 (1.0)	10.5 (4.3)	49.8 (11.1)	1.2 (1.2)	62.7 (4.7)
		CI	97.7 (1.7)	14.2 (6.8)	87.1 (3.1)	76.0 (2.7)	93.7 (1.9)	34.0 (5.5)	73.7 (1.2)	32.2 (11.3)	92.5 (2.5)	27.2 (8.2)	91.0 (1.4)
		P_g_	5	1	4	4	5	2	4	2	4	2	5
R3598	1	SCO_max_	1.3 (0.6)	0.0 (0.0)	0.0 (0.0)	0.0 (0.0)	0.6 (0.3)	0.0 (0.0)	0.1 (0.0)	0.1 (0.1)	0.0 (0.0)	0.0 (0.0)	0.6 (0.6)
		W_a_	11.4 (6.2)	0.0 (0.0)	0.0 (0.0)	0.4 (0.4)	4.9 (2.7)	0.0 (0.0)	0.5 (0.1)	2.4 (2.4)	0.2 (0.2)	0.0 (0.0)	3.9 (3.9)
		CI	77.8 (15.8)	2.0 (2.0)	5.0 (5.0)	21.0 (3.0)	61.0 (5.0)	4.0 (1.0)	16.3 (1.1)	2.6 (2.6)	40.0 (0.0)	0.0 (0.0)	74.7 (14.7)
		P_g_	3.2	1	1	2	3.2	1	1	1	2	1	3.2
	2	SCO_max_	1.6 (0.4)	0.1 (0)	0.0 (0.0)	0.4 (0.2)	0.4 (0.1)	0.0 (0.0)	0.1 (0.0)	0.1 (0.1)	0.0 (0.0)	0.1 (0.1)	0.4 (0.3)
		W_a_	11.5 (3.1)	0.2 (0.2)	0.0 (0.0)	2.4 (1.2)	2.5 (0.7)	0.0 (0.0)	0.4 (0.1)	0.9 (0.9)	0.1 (0.1)	0.7 (0.7)	3.3 (2.2)
		CI	92.0 (6.0)	9.0 (3.0)	10.0 (0.0)	61.0 (8.0)	81.0 (8.0)	6.0 (1.0)	12.1 (1.3)	27.5 (7.5)	20.0 (5.0)	10.0 (0.0)	72.5 (12.5)
		P_g_	4	1	1	3.2	3.2	1	1	2	2	1	3.2
	Comb	SCO_max_	1.5 (0.3)	0.0 (0.0)	0.0 (0.0)	0.2 (0.1)	0.5 (0.2)	0.0 (0.0)	0.1 (0.0)	0.1 (0.1)	0.0 (0.0)	0.0 (0.0)	0.5 (0.3)
		W_a_	11.4 (2.8)	0.1 (0.1)	0.0 (0.0)	1.4 (0.8)	3.7 (1.3)	0.0 (0.0)	0.5 (0.1)	1.6 (1.1)	0.2 (0.1)	0.3 (0.3)	3.6 (1.8)
		CI	84.9 (8.0)	5.5 (2.5)	7.5 (2.5)	41.0 (12.1)	71.0 (6.9)	5.0 (0.8)	16.3 (1.1)	15.1 (7.9)	30.0 (6.1)	5.0 (2.9)	73.6 (7.9)
		P_g_	4	1	1	2	3.2	1	1	1	2	1	3.2

^a^ Variables presented include the maximal score (SCO_max_) and the area under disease progress curve for incidence (W_a_), expressed as a percentage, and the colonization index (CI), expressed as a percentage. The phenotypic groups (P_g_) were estimated as described in Lebeau et al. [26].

**Table 2:** Sources of phenotypic variation (Anova analysis) and estimates of heritabilities (broad sense) for SCO_max_, W_a_, and CI for the EG203 × MM738 DH population.

Strain	Season	Var ^a^	Sources of Variations ^b^				h^2^ (Interval) ^c^
			G	S	R	G × S	
PSS4	1	SCO_max_	***	-	ns	-	0.68 (0.56–0.79)
		W_a_	***	-	ns	-	0.70 (0.59–0.80)
		CI	***	-	**	-	0.34 (0.23–0.45)
	2	SCO_max_	***	-	ns	-	0.57 (0.41–0.72)
		W_a_	***	-	ns	-	0.58 (0.42–0.72)
		CI	***	-	**	-	0.36 (0.23–0.49)
	Comb	SCO_max_	***	***	ns	**	0.71 (0.59–0.80)
		W_a_	***	***	ns	**	0.70 (0.57–0.80)
		CI	***	***	***	*	0.50 (0.40–0.60)
R3598	1	SCO_max_	-	-	-	-	-
		W_a_	-	-	-	-	-
		CI	***	-	ns	-	0.53 (0.36–0.68)
	2	SCO_max_	-	-	-	-	-
		W_a_	-	-	-	-	-
		CI	***	-	**	-	0.56 (0.41–0.69)
	Comb	SCO_max_	-	-	-	-	-
		W_a_	-	-	-	-	-
		CI	***	***	*	**	0.39 (0.26–0.53)

^a^ The variables presented include the maximal score (SCO_max_), the area under disease progress curve for wilting incidence (W_a_), expressed as a percentage, and the colonization index (CI), expressed as apercentage. ^b^ Effects included in Anova: genotype effect (G), season effect (S), repetition effect (R), and interaction between genotype and season (G × S). A linear model was used on SCO_max_, and a linear mixed model was used on W_a_, assuming a normal distribution with strain PSS4. A generalized linear model was used for CI, assuming a binomial distribution with both strains. In the mixed model, the S and R effects were considered as fixed, whereas the G and G × S effects were considered random. ^c^ Broad-sense heritabilities and their 95% Bayesian confidence interval were estimated; *, **, ***: significant at *p* < 0.05, *p* < 0.01, and *p* < 0.001, respectively; ns: Not significant; -: Not estimated.

**Table 3:** QTLs of resistance to PSS4 and R3598 strains, detected by multiple QTL mapping (stepwise model) for the EG203 × MM738 DH population, on individual seasons and across the seasons (Comb).

Strains	Season	Var ^a^	Chr ^b^	QTL ^c^	Pos ^d^	Nearest Marker	Interval ^e^	LOD	R^2 f^	Add Effect ^g^	Total R^2 h^
PSS4	1	SCO_max_	E03	*ERPR3a*	16.0	s296164	3.0–29.0	3.8	13.2	−0.38 ***	13.2
		W_a_	E03	*ERPR3a*	16.0	s296164	2.0–27.2	3.4	11.9	−9.11 ***	11.9
		CI	E01	*ERPR1*	76.8	s219212	47.0–88.3	3.5	4.8	−4.96 ***	65.4
			E02	*ERPR2a*	40.0	s135116	34.0–77.0	4.1	5.7	−5.55 ***	
			E03	*ERPR3a*	4.1	s227496	0.0–13.0	7.3	10.8	−7.69 ***	
			E03	*ERPR3b*	126.7	s231411	125.8–140.0	10.7	17.0	−9.49 ***	
			E04	*ERPR4*	101.3	s46892	99.0–102.0	8.5	13.0	−7.23 ***	
			E06	*ERPR6a*	23.0	s902	22.0–33.0	10.2	16.0	−7.32 ***	
			E07	*ERPR7*	41.6	s272460	35.0–45.0	6.4	9.4	−6.87 ***	
			Epistasis	*ERPR4:ERPR6a*				2.9	4.0	−4.65 ***	
	2	SCO_max_	E02	*ERPR2b*	98.4	s51429	94.0–104.0	5.5	12.3	−0.33 ***	45.7
			E04	*ERPR4*	79.0	s400	68.0–92.0	5.0	11.2	−0.32 ***	
			E06	*ERPR6b*	40.0	s431	38.0–43.0	9.9	25.3	−0.48 ***	
			E08	*ERPR8*	23.4	s105566	18.0–28.6	5.0	11.5	−0.31 ***	
		W_a_	E02	*ERPR2b*	98.4	s51429	94.0–103.0	5.7	13.8	−3.86 ***	40.4
			E04	*ERPR4*	88.4	s208766	74.0–105.0	4.2	8.0	−2.93 ***	
			E06	*ERPR6b*	41.0	s147256	38.6–49.0	7.5	16.8	−4.41 ***	
			E08	*ERPR8*	23.4	s105566	18.0–54.0	4.7	11.0	−3.50 ***	
		CI	E01	*ERPR1*	38.8	s231295	19.0–109.0	3.1	10.9	−5.83 ***	10.9
	Comb	SCO_max_	E02	*ERPR2b*	98.4	s51429	82.0–102.2	6.2	12.1	−0.30 ***	54.0
			E03	*ERPR3a*	21.0	s168838	2.0–31.0	3.5	6.4	−0.23 ***	
			E04	*ERPR4*	71.3	s53085	66.0–97.0	5.5	10.5	−0.29 ***	
			E06	*ERPR6b*	40.0	s431	39.0–42.2	10.3	21.7	−0.43 ***	
			E08	*ERPR8*	23.4	s105566	18.0–28.6	5.5	10.4	−0.28 ***	
		W_a_	E02	*ERPR2b*	98.4	s51429	79.0–102.2	5.0	11.4	−3.29 ***	44.9
			E04	*ERPR4*	97.0	s311121	68.0–107.0	4.8	10.9	−3.26 ***	
			E06	*ERPR6b*	40.3	s147256	39.0–43.0	7.7	18.5	−4.28 ***	
			E08	*ERPR8*	23.4	s105566	17.0–50.0	4.6	10.4	−3.17 ***	
		CI	E01	*ERPR1*	68.0	s168619	43.3–84.2	4.5	7.9	−4.98 ***	56.9
			E02	*ERPR2b*	87.7	s131841	74.0–99.2	5.0	8.9	−5.13 ***	
			E03	*ERPR3a*	4.1	s227496	2.0–27.0	4.5	7.8	−4.98 ***	
			E03	*ERPR3b*	127.5	s231125	124.0–130.0	7.1	13.1	−6.37 ***	
			E04	*ERPR4*	71.0	s53085	66.6–79.6	6.6	12.0	−6.22 ***	
			E06	*ERPR6a*	39.4	s56388	26.0–45.0	6.5	11.9	−6.19 ***	
R3598	1	SCO_b_	E03	*ERPR3a*	4.7	s231544	0.0–28.0	3.2	11.4	−1.09 ***	11.4
		CI_10b_	E03	*ERPR3a*	5.0	s197676	2.0–7.2	9.4	29.7	−1.34 ***	29.7
		CI_b_	E03	*ERPR3a*	5.0	s197676	3.0–7.2	7.3	21.4	−1.39 ***	31.3
			E06	*ERPR6c*	46.0	s681	39.0–65.0	4.3	12.0	−1.06 ***	
	2	SCO_b_	ns	ns	ns	ns	ns	ns	ns	ns	ns
		CI_10b_	E09	*ERPR9*	51.5	s75856	42.9–59.1	4.4	15.0	−0.95 ***	15.0
		CI_b_	ns	ns	ns	ns	ns	ns	ns	ns	ns
	Comb	SCO.b	ns	ns	ns	ns	ns	ns	ns	ns	ns
		CI10.b	E03	*ERPR3a*	4.1	s227496	0.0–7.0	8.0	25.8	−1.20 ***	25.8
		CI.b	E03	*ERPR3a*	5.0	s197676	0.0–14.0	4.0	12.6	−1.04 ***	21.6
			E06	*ERPR6c*	46.0	s681	9.9–70.2	3.3	10.3	−0.91 ***	

^a^ The variables used are the maximal score (SCO_max_), the BLUP of area under disease progress curve for wilting percentage (Wa) and the colonization index (CI). ^b^ Linkage groups named according to their chromosome correspondence. ^c^ Name of the QTL: Eggplant *Ralstonia Pseudosolanacearum* Resistance (ERPR) followed by the chromosome number. When several QTLs were detected on the same chromosome, a letter follows the QTL name for identification purposes. ^d^ Position of the maximum logarithm of odds score (LOD) in centimorgans (cM). ^e^ 95% Bayesian confidence interval (cM). ^f^ Estimates of the percentage of phenotypic variance explained by the QTL detected. ^g^ Additive effect: a positive value indicates that the resistance comes from the E8 (MM738) allele, a negative value indicates that the resistance comes from the E4 (EG203) allele. ^h^ Estimates of the total percentage of phenotypic variance explained by the additive model; ns: QTLs not detected above the LOD threshold. R^2^;***: significant at *p* < 0.001.

**Table 4:** Digenic interactions for the pairs of loci *ERPR2b/ERPR6b*, *ERPR2b/ERPR8*, *ERPR3a/ERPR6b*, *ERPR3a/ERPR8*, *ERPR4/ERPR6b*, and *ERPR4/ERPR8.* Mean of SCO_max_ ± standard deviation is indicated for each pair of loci. The groups defined by the least significant difference (LSD), just after means and standard deviations. The number of DH lines per genotype class is indicated in parenthesis. “A”: E8 (MM738) allele and “B”: E4 (EG203) allele.

Pair of Loci	Genotypes at the First and Second Loci			
	AA/AA	AA/BB	BB/AA	BB/BB
*ERPR2b/ERPR6b*	2.72 ± 0.67 a (34)	2.24 ± 0.83 a (32)	2.31 ± 0.65 a (35)	1.40 ± 0.83 b (22)
*ERPR2b/ERPR8*	2.72 ± 0.78 a (37)	2.19 ± 0.69 b (29)	2.09 ± 0.89 b (29)	1.83 ± 0.79 b (28)
*ERPR3a/ERPR6b*	2.60 ± 0.68 a (37)	2.39 ± 0.96 a (21)	2.41 ± 0.67 a (33)	1.57 ± 0.76 b (32)
*ERPR3a/ERPR8*	2.82 ± 0.68 a (29)	2.23 ± 0.80 b (29)	2.15 ± 0.91 b (37)	1.79 ± 0.65 b (28)
*ERPR4/ERPR6b*	2.80 ± 0.65 a (26)	2.22 ± 0.84 ab (32)	2.34 ± 0.64 b (44)	1.39 ± 0.83 c (21)
*ERPR4/ERPR8*	2.73 ± 0.78 a (33)	2.15 ± 0.73 b (25)	2.15 ± 0.88 b (33)	1.90 ± 0.77 b (32)

a; ab; b; c: groups defined by the least significant difference (LSD) test.

**Table 5:** Bacterial Wilt-resistance QTLs detected in the DH population EG203 × MM738 for which a correspondence with published Solanaceae BW-resistance QTLs was found.

Summary of QTLs Detected with PSS4 and R3598 Strains				BW-Resistance QTLs in Solanaceae Crops					
Name	Interval (cM)	Position ^a^	Strain	Name ^b^	Species	Cultivar	Position ^a^	Strains ^c^	Studies ^d^
*ERPR2a*	34–77	37.6–43.9	PSS4	*EBWR2*	Eggplant	AG91-25	38.3–46.9	PSS4, TO10, CFPB2957, CFBP3059	8
*ERPR2b*	74–104	43.5–53.1	PSS4						
*ERPR3b*	124–139	65.2–67.8	PSS4	*Bwr-3*	Tomato	Hawaii 7996	61.2–70.8	GMI8217, JT516, Tm151	2,6,7
*ERPR6a*	22–45	0.0–37.8	PSS4	*Bwr-6*	Tomato	Hawaii 7996, L285	36.9–38.8	PSS4, PSS186, Tm151, JT519, UW364, JT516, GMI8217	1,2,3,4,5,6,7
*ERPR6b*	38–49	25.7–38.9	PSS4						
*ERPR6c*	39–65	25.7–41.9	R3598						

^a^ Position on tomato genome in Mb. ^b^ Published QTL name. *EBWR2* for eggplant bacterial wilt resistance on chr 2 and *Bwr* following chr number for bacterial wilt resistance. ^c^ Strains used in the previous studies: PSS4, TO10, Pss186, Tm151, JT519, and UW364 belong to phylotype I; CFBP2957, JT516, and GMI8217 belong to phylotype II; CFBP3059 belong to phylotype III. **^d^** Reference: (1) [42]; (2) [23]; (3) [24]; (4) [22]; (5) [25]; (6) [21]; (7) [20] and (8) [32].

**Table 6:** Description of the location, period of assay, strain used for the inoculation, and mean Temperature (T (°C)) and Relative Humidity (RH (%)) (with standard errors) measured during the phenotyping assays conducted on the DH population EG203 × MM738.

Location	Season	Period of Assay	Strain	T (°C)	RH (%)
Reunion Island	1	November 2016	PSS4	27 (±8)	73 (±23)
	2	May 2016	PSS4	25 (±7)	82 (±21)
Cameroon	1	March 2016	R3598	32 (±10)	66 (±28)
	2	June 2016	R3598	28 (±7)	74 (±23)

## Supplementary Materials

Supplementary materials can be found at http://www.mdpi.com/1422-0067/19/2/357/s1.
