# Supplementary material for: Genotyping by Sequencing Highlights a Polygenic Resistance to Ralstonia pseudosolanacearum in Eggplant (Solanum melongena L.)
Source: Int J Mol Sci. 2018 Jan 25;19(2):357. doi: 10.3390/ijms19020357 (PMC5855579; doi:10.3390/ijms19020357)
Supplement: Supplementary file 1 [file ijms-19-00357-s001.zip › ijms-261347 supplementary/ijms-261347 supplementary.pdf]

Table S1. List of DNA samples used for genotyping by sequencing (GBS). Their associated barcode sequences and the number of reads after demultiplexing and cleaning steps are indicated.

Individuals	Type	Sequencing lane	Barcodes	Number of reads	Comments
MM738	E8	1	TTGTT/TTGACGT	3,462,252	
EG203	E4	1	TTCAGA/TTGCAGGA	6,632,870	
S001	DH	1	AACT/GGCTGA	8,497,078	
S002	DH	1	CCTA/CAAGTT	4,203,095	
S003	DH	1	TTAC/TGTTCA	6,003,998	
S004	DH	1	AGGC/TTGCAGA	6,826,164	
S005	DH	1	GCAT/AACAGGT	5,038,625	
S006	DH	1	TAGA/CCACTGA	7,136,508	
S007	DH	1	GTCA/CTTGAGT	7,694,123	
S008	DH	1	GATC/TGCTGGA	5,614,369	
S010	DH	1	CTGT/GAGCAGT	5,986,442	
S011	DH	1	AGAGA/ACAACGT	2,005,225	
S012	DH	1	CGCGT/CTGGTGA	1,778,691	
S013	DH	1	TCTGA/GCTTGGA	3,909,145	
S014	DH	1	AACAT/TGACAGT	3,120,711	
S015	DH	1	CCACA/AACGTGA	3,351,868	
S016	DH	1	GATGT/CATTGGT	2,687,729	
S017	DH	1	CGTAA/GGCCTGA	5,029,519	
S018	DH	1	ACATT/ACTAAGA	4,419,904	
S019	DH	1	TTCCA/TTAGCTT	8,540,098	
S020	DH	1	GTGGA/CGGTGAA	4,673,236	
S021	DH	1	AGTCT/GAATCGT	3,395,762	
S022	DH	1	CACTA/ACCAGTA	7,270,665	
S023	DH	1	TGAAT/TTGCTAT	5,312,278	
S024	DH	1	GCGAT/GGTGAGC	4,810,672	
S025	DH	1	AAGCA/AACAATGA	6,288,181	
S026	DH	1	CTAGC/CCACCGGT	2,632,355	
S027	DH	1	TCCTC/CTTGTAGT	3,439,552	
S028	DH	1	GTACT/TCGTGTGA	3,232,965	
S029	DH	1	GGTTC/GGAACGGT	4,262,172	
S030	DH	1	ACGGC/AATCAAGA	3,773,710	discarded
S031	DH	1	CACAGT/TTCGTTGA	5,536,563	
S032	DH	1	TCTGGA/CGGTGAGT	5,797,771	
S033	DH	1	AGACGT/GAATCGGA	5,574,131	
S034	DH	1	CATTGA/ACCATTGT	3,347,110	
S035	DH	1	GCAGGT/GCTGGAGT	4,716,677	
S036	DH	1	TGGCGA/AGACCTTA	5,118,805	
S037	DH	1	ATCGGT/CACAAGAT	3,388,453	
S038	DH	1	CAGTAT/TGTTGCGA	3,911,296	
S039	DH	1	CGTATA/CTGGTTAT	3,031,826	
S041	DH	1	GAGCGT/GAACAATA	3,017,599	
S042	DH	1	ACATGA/ACCACGCT	3,637,223	
S043	DH	1	TGCAAT/GTTGTCGA	8,570,216	
S044	DH	1	CTTGAA/TCGTGATT	4,245,058	
S045	DH	1	GACATA/AGACTTAT	4,464,119	
S046	DH	1	ACTAGT/CACTCCGA	6,748,737	
blank	water	1	GTACAA/ACGGTT/TTGAAGCT/GGTGGCAA	2,344	

Table S1. Continued

Individuals	Type	Sequencing lane	Barcodes	Number of reads	Comments
MM738	E8	2	GCAGGT/GCTGGAGT	10,532,051	
EG203	E4	2	CGCGT/CTGGTGA	7,793,210	
S047	DH	2	AACT/GGCTGA	13,789,177	
S048	DH	2	CCTA/CAAGTT	13,834,948	
S049	DH	2	TTAC/TGTTCA	11,133,247	
S050	DH	2	AGGC/TTGCAGA	9,924,802	
S051	DH	2	GCAT/AACAGGT	11,456,666	
S052	DH	2	TAGA/CCACTGA	10,694,160	discarded
S053	DH	2	GTCA/CTTGAGT	17,547,472	discarded
S054	DH	2	GATC/TGCTGGA	10,387,132	
S055	DH	2	CTGT/GAGCAGT	9,846,556	
S056	DH	2	AGAGA/ACAACGT	8,501,194	
S057	DH	2	TCTGA/GCTTGGA	10,032,536	
S058	DH	2	AACAT/TGACAGT	7,823,310	
S059	DH	2	CCACA/AACGTGA	9,845,834	
S060	DH	2	TTGTT/TTGACGT	12,652,931	
S061	DH	2	GATGT/CATTGGT	10,721,515	
S062	DH	2	CGTAA/GGCCTGA	13,784,824	
S063	DH	2	ACATT/ACTAAGA	15,498,404	
S064	DH	2	TTCCA/TTAGCTT	19,421,485	
S065	DH	2	GTGGA/CGGTGAA	15,415,339	
S066	DH	2	AGTCT/GAATCGT	12,782,101	
S067	DH	2	CACTA/ACCAGTA	19,502,579	
S068	DH	2	TGAAT/TTGCTAT	15,809,648	
S069	DH	2	GCGAT/GGTGAGC	14,272,550	
S070	DH	2	AAGCA/AACAATGA	13,409,675	
S071	DH	2	CTAGC/CCACCGGT	6,346,932	
S072	DH	2	TCCTC/CTTGTAGT	11,771,704	
S073	DH	2	GTACT/TCGTGTGA	10,207,550	
S074	DH	2	GGTTC/GGAACGGT	13,080,967	
S075	DH	2	ACGGC/AATCAAGA	18,410,703	
S076	DH	2	CACAGT/TTCGTTGA	12,020,621	
S077	DH	2	TCTGGA/CGGTGAGT	19,137,029	
S078	DH	2	AGACGT/GAATCGGA	17,542,719	
S079	DH	2	CATTGA/ACCATTGT	17,961,161	
S080	DH	2	TTCAGA/TTGCAGGA	18,069,543	
S081	DH	2	TGGCGA/AGACCTTA	16,883,692	
S083	DH	2	ATCGGT/CACAAGAT	14,448,301	
S084	DH	2	CAGTAT/TGTTGCGA	15,793,865	
S085	DH	2	CGTATA/CTGGTTAT	13,429,486	
S086	DH	2	GAGCGT/GTTGTCGA	17,187,924	
S087	DH	2	ACATGA/TCGTGATT	11,366,228	
S088	DH	2	TGCAAT/AGACTTAT	11,032,107	
S089	DH	2	CTTGAA/CACTCCGA	15,615,216	
S090	DH	2	GACATA/TTGAAGCT	17,983,771	discarded
S091	DH	2	ACTAGT/GGTGGCAA	15,994,180	
Blank	water	2	GTACAA/ACGGTT/GAACAATA/AC	12,238	

Table S1. Continued

Individuals	Type	Sequencing lane	Barcodes	Number of reads	Comments
**MM738**	E8	3	GTACT/TCGTGTGA	6,034,126	
**EG203**	E4	3	TTCCA/TTAGCTT	8,568,806	
**S093**	DH	3	AACT/GGCTGA	18,965,064	
**S094**	DH	3	CCTA/CAAGTT	12,585,405	
**S095**	DH	3	TTAC/TGTTCA	14,228,325	
**S096**	DH	3	AGGC/TTGCAGA	14,226,544	
**S097**	DH	3	GCAT/AACAGGT	12,919,922	
**S098**	DH	3	TAGA/CCACTGA	11,952,642	
**S099**	DH	3	GTCA/CTTGAGT	10,399,336	discarded
**S100**	DH	3	GATC/TGCTGGA	13,575,317	
**S101**	DH	3	CTGT/GAGCAGT	8,560,256	
**S102**	DH	3	AGAGA/ACAACGT	4,565,124	
**S103**	DH	3	CGCGT/CTGGTGA	8,160,185	
**S104**	DH	3	TCTGA/GCTTGGA	9,527,121	
**S105**	DH	3	AACAT/TGACAGT	8,177,073	
**S106**	DH	3	CCACA/AACGTGA	9,272,738	discarded
**S107**	DH	3	TTGTT/TTGACGT	7,188,519	
**S108**	DH	3	GATGT/CATTGGT	4,861,281	
**S109**	DH	3	CGTAA/GGCCTGA	12,411,774	
**S110**	DH	3	ACATT/ACTAAGA	11,997,503	
**S112**	DH	3	GTGGA/CGGTGAA	13,438,920	
**S113**	DH	3	AGTCT/GAATCGT	10,681,632	discarded
**S115**	DH	3	CACTA/ACCAGTA	11,856,418	discarded
**S116**	DH	3	TGAAT/TTGCTAT	10,864,011	discarded
**S117**	DH	3	GCGAT/GGTGAGC	9,592,038	
**S118**	DH	3	AAGCA/AACAATGA	14,275,060	
**S119**	DH	3	CTAGC/CCACCGGT	5,746,406	
**S120**	DH	3	TCCTC/CTTGTAGT	7,946,874	
**S121**	DH	3	GGTTC/GGAACGGT	10,724,441	
**S122**	DH	3	ACGGC/AATCAAGA	10,198,397	
**S123**	DH	3	CACAGT/TTCGTTGA	12,139,396	
**S124**	DH	3	TCTGGA/CGGTGAGT	16,931,721	
**S125**	DH	3	AGACGT/GAATCGGA	16,533,100	
**S126**	DH	3	CATTGA/ACCATTGT	12,048,671	
**S127**	DH	3	TTCAGA/TTGCAGGA	19,618,234	
**S128**	DH	3	GCAGGT/GCTGGAGT	9,732,280	
**S129**	DH	3	TGGCGA/AGACCTTA	12,414,933	
**S132**	DH	3	ATCGGT/CACAAGAT	11,479,474	
**S134**	DH	3	CAGTAT/TGTTGCGA	12,038,141	
**S135**	DH	3	CGTATA/CTGGTTAT	6,796,486	
**S137**	DH	3	GAGCGT/ACCACGCT	8,317,235	
**S141**	DH	3	ACATGA/GTTGTCGA	16,591,861	
**S143**	DH	3	TGCAAT/TCGTGATT	7,506,787	
**S148**	DH	3	CTTGAA/AGACTTAT	7,841,993	
**S166**	DH	3	GACATA/CACTCCGA	13,114,234	
**S177**	DH	3	ACTAGT/TTGAAGCT	10,799,633	
**Blank**	water	3	GTACAA/ACGGTT/GAACAATA/GGTGGCAA	8,657	

Table S2. Descriptive statistics of the newly developed [EG203 x MM738] genetic map.

Linkage group (LG)	Number of SNPs	Average interval[a] (cM)	Maximum interval[b] (cM)	Length (cM)	% Skewed markers[c]	Estimated genome length (cM)	Genome Coverage (%)
E01	141	1.07	8.14	150.85	4.96	153.01	86.08
E02	114	1.28	9.75	146.13	3.51	148.72	85.95
E03	132	1.27	9.10	167.34	12.12	169.89	86.10
E04	91	1.33	8.32	120.90	1.10	123.59	85.89
E05	79	1.26	7.53	99.91	0.00	102.47	85.67
E06	107	1.08	6.60	115.23	10.28	117.40	86.04
E07	95	0.99	5.30	94.44	0.00	96.45	85.78
E08	88	1.31	11.32	114.93	12.50	117.57	85.93
E09	90	1.35	34.90	121.13	7.78	123.85	85.94
E10	83	1.44	9.13	119.24	28.92	122.15	85.87
E11	97	1.23	10.27	119.63	0.0	122.12	85.83
E12	53	1.72	15.11	91.39	11.32	94.91	85.35
Total	1170	1.25	34.90	1461.02	7.44	1492.13	85.92

[a] Mean number of markers per cM.

[b] Maximum interval without markers.

[c] Percentage of markers, which deviate from the 1:1 expected segregation ratio in a DH population at P-value < 0.05.

Table S3. Excel file with the complete genetic map of [EG203 × MM738] DH population and SNP sequences' significant hits on eggplant, tomato, pepper and potato.

Table S4. Pearson correlation coefficients among variables measured during two seasons and computed across seasons (Comb) in the DH population inoculated with strain R3598 (Cameroon assay).

Season		1					2					Comb				
	Var[a]	SCO_{max}	W_{max}	SCO_a	W_a	CI	SCO_{max}	W_{max}	SCO_a	W_a	CI	SCO_{max}	W_{max}	SCO_a	W_a	CI
1	SCO_{max}	1.00														
	W_{max}	0.99	1.00													
	SCO_a	0.86	0.83	1.00												
	W_a	0.88	0.86	0.99	1.00											
	CI	0.61	0.62	0.57	0.59	1.00										
2	SCO_{max}	0.33	0.32	0.26	0.28	0.31	1.00									
	W_{max}	0.35	0.34	0.28	0.31	0.33	0.93	1.00								
	SCO_a	0.26	0.24	0.22	0.22	0.27	0.97	0.82	1.00							
	W_a	0.29	0.27	0.24	0.24	0.28	0.97	0.88	0.98	1.00						
	CI	0.50	0.49	0.39	0.40	0.50	0.71	0.68	0.66	0.69	1.00					
Comb	SCO_{max}	0.91	0.89	0.78	0.80	0.61	0.69	0.67	0.62	0.65	0.69	1.00				
	W_{max}	0.82	0.83	0.69	0.72	0.58	0.75	0.81	0.64	0.69	0.72	0.96	1.00			
	SCO_a	0.86	0.82	0.97	0.97	0.59	0.48	0.46	0.45	0.46	0.52	0.87	0.79	1.00		
	W_a	0.84	0.81	0.91	0.92	0.59	0.62	0.60	0.57	0.60	0.60	0.92	0.87	0.98	1.00	
	CI	0.65	0.65	0.57	0.58	0.89	0.57	0.56	0.51	0.53	0.84	0.75	0.74	0.64	0.69	1.00

[a] Variables measured: the maximal score (SCO_{max}); the maximal wilting percentage (W_{max}); the of area under disease progress curve for the score (SCO_a); the wilting percentage (W_a) and the colonization percentage (CI).

Table S5. Pearson correlation coefficients among variables measured during two seasons and computed across seasons (Comb) in the DH population inoculated with strain PSS4 (Reunion assay).

Season		1					2					Comb				
	Var[a]	SCO_{max}	W_{max}	SCO_a[b]	W_a[b]	CI	SCO_{max}	W_{max}	SCO_a[b]	W_a[b]	CI	SCO_{max}	W_{max}	SCO_a[b]	W_a[b]	CI
1	SCO_{max}	1.00														
	W_{max}	0.98	1.00													
	SCO_a[b]	0.98	0.96	1.00												
	W_a[b]	0.98	0.97	0.99	1.00											
	CI	0.86	0.88	0.82	0.84	1.00										
2	SCO_{max}	0.50	0.49	0.47	0.47	0.55	1.00									
	W_{max}	0.50	0.49	0.47	0.47	0.54	0.99	1.00								
	SCO_a[b]	0.49	0.48	0.47	0.47	0.54	0.95	0.91	1.00							
	W_a[b]	0.49	0.48	0.47	0.48	0.54	0.95	0.93	0.99	1.00						
	CI	0.47	0.47	0.43	0.43	0.50	0.79	0.81	0.71	0.72	1.00					
Comb	SCO_{max}	0.88	0.87	0.86	0.86	0.82	0.85	0.84	0.81	0.82	0.72	1.00				
	W_{max}	0.88	0.88	0.85	0.86	0.83	0.83	0.84	0.78	0.79	0.73	0.99	1.00			
	SCO_a[b]	0.85	0.84	0.86	0.85	0.79	0.83	0.81	0.86	0.86	0.67	0.97	0.95	1.00		
	W_a[b]	0.86	0.85	0.85	0.86	0.81	0.83	0.81	0.85	0.86	0.67	0.97	0.96	0.99	1.00	
	CI	0.79	0.80	0.74	0.76	0.89	0.76	0.76	0.71	0.71	0.83	0.90	0.91	0.85	0.86	1.00

[a] Variables measured: the maximal score (SCO_{max}); the maximal wilting percentage (W_{max}); the area under disease progress curve for the score (SCO_a); the wilting percentage (W_a) and the colonization percentage (CI).

[b] Best linear unbiased predictor (Blup) of the SCO_a and W_a variables were computed for trials with PSS4 strain.

Table S6. QTLs of resistance to PSS4 strain (Reunion assay), detected by Simple Interval Mapping and Haley-Knott regression model, assuming a normal distribution in the [EG203 x MM738] DH population. QTL analysis was carried out for individual seasons, as well as for combined seasons (Comb).

Season	Var[a]	Chr[b]	QTL[c]	Pos[d]	Nearest marker	Interval[e]	LOD	R^{2f}	Add effect[g]	Total R^{2h}
1	SCO_{max}	E03	*ERPR3a*	16.0	s296164	3.0-28.0	3.8	13.2	-0.38***	13.2
	W_a	E03	*ERPR3a*	16.0	s296164	0.0-167.3	3.0	9.3	-3.36**	20.4
		E04	*ERPR4*	98.0	s311121	93.0-110.0	3.6	11.7	-3.97***	
		Epistasis		*ERPR3a:ERPR4*			1.0	3.1	2.30*	
	CI	ns	ns	ns	ns	ns	ns	ns	ns	ns
2	SCO_{max}	E06	*ERPR6b*	40.2	s431	6.0-52.0	3.6	12.5	-0.33***	12.5
	W_a	E02	*ERPR2b*	98.4	s51429	84.0-114.0	3.4	11.9	-3.56***	11.9
	CI	E01	*ERPR1*	38.8	s231295	19.0-108.0	3.1	10.9	-5.83***	10.9
Comb	SCO_{max}	E03	*ERPR3a*	16.0	s296164	3.7-167.3	4.4	11.5	-0.24***	35.7
		E04	*ERPR4*	103.8	s298545	66.0-113.0	4.6	12.1	-0.23***	
		E06	*ERPR6b*	42.0	s11362	39.0-44.0	7.0	19.2	-0.22***	
		Epistasis		*ERPR3a:ERPR4*			1.4	3.6	-0.06*	
				ERPR3a:ERPR6b			3.0	7.7	-0.18**	
				ERPR4:ERPR6b			1.4	3.5	0.02*	
				ERPR3a:ERPR4:ERPR6b			1.4	3.5	-0.17*	
	W_a	E02	*ERPR2b*	95.0	s116247	72.0-118.0	4.4	14.0	-3.07***	22.1
		E04	*ERPR4*	91.0	s260898	85.0-113.0	3.5	10.9	-2.85***	
		Epistasis		*ERPR2b:ERPR4*			1.1	3.2	-1.80*	
	CI	E03	*ERPR3b*	126.7	s231411	4.0-167.3	3.7	12.8	-6.16***	12.8

[a] The variables used are maximal score (SCOmax), BLUP of area under disease curve progress for wilting percentage (Wa) and colonization percentage (CI).

[b] Linkage groups named according to their chromosome correspondence.

[c] Name of the QTL: Eggplant *Ralstonia pseudosolanacearum* Resistance (*ERPR*) followed by the chromosome number. When several QTLs are detected on the same chromosome, a letter is added to the QTL name for identification purposes.

[d] Position of the maximum logarithm of odds score (LOD) in centimorgans (cM).

[e] 95% Bayesian confidence interval (cM).

[f] Estimates of the percentage of phenotypic variance explained by the QTL detected.

[g] Additive effect: a positive value indicates that resistance comes from the E8 (MM738) allele, while a negative value indicates that resistance comes from the E4 (EG203) allele.

[h] Estimate of the total percentage of phenotypic variance explained by the additive model.

ns: QTLs were not detected above the LOD threshold.

Table S7. QTLs of resistance to R3598 strain (Cameroon assay), detected by simple interval mapping, and a non-parametric model for the [EG203 x MM738] DH population. QTL analysis was carried out for individual seasons, as well as for combined seasons (Comb).

Season	Var[a]	Chr[b]	QTL[c]	Pos[d]	Nearest marker	Interval[e]	LOD
1	SCO_{max}	E03	*ERPR3a*	4.7	s231544	0.0-28.0	3.1
	W_a	E03	*ERPR3a*	4.7	s231544	0.0-28.0	3.2
	CI	E03	*ERPR3a*	5.0	s197676	2.0-6.4	9.7
2	SCO_{max}	E09	*ERPR9*	51.5	s75856	40.0-80.7	2.9
	W_a	ns	ns	ns	ns	ns	ns
	CI	E09	*ERPR9*	49.4	s298980	42.0-67.0	3.2
Comb	SCO_{max}	ns	ns	ns	ns	ns	ns
	W_a	ns	ns	ns	ns	ns	ns
	CI	E03	*ERPR3a*	4.1	s227496	0.0-6.4	7.7

[a] Variables used are the maximal score (SCO_{max}), the area under disease curve progress for wilting percentage (W_a) and the colonization percentage (CI).

[b] Linkage groups are named according to their chromosome correspondence.

[c] Name of the QTL: Eggplant *Ralstonia pseudosolanacearum* Resistance (*ERPR*) followed by the chromosome number. When several QTLs are detected on the same chromosome, they are identified by a different letter after the QTL name.

[d] Position of the maximum logarithm of odds score (LOD) in centimorgans (cM).

[e] 95 % Bayesian confidence interval (cM).

ns: QTLs were not detected above the LOD threshold.

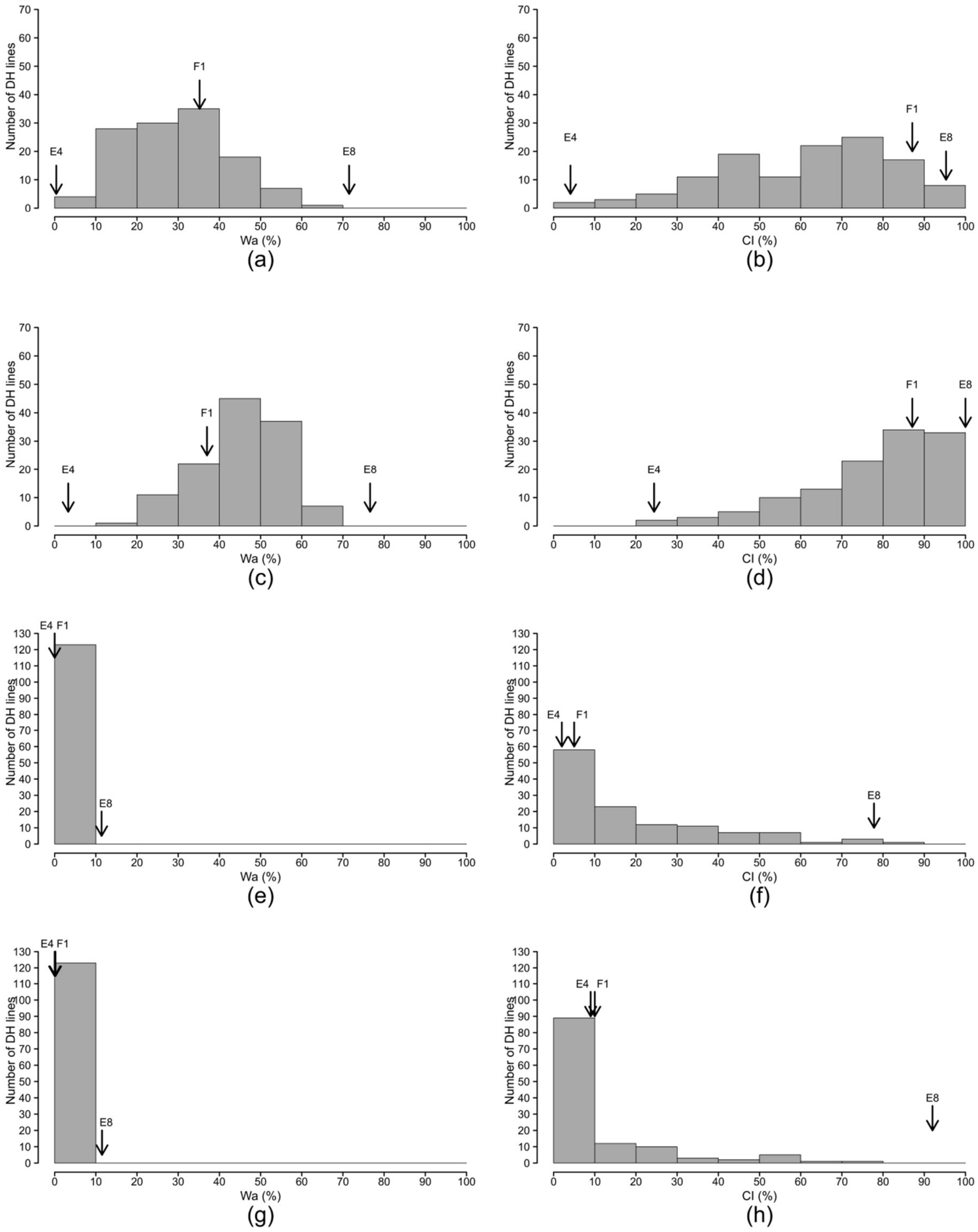

Figure S1. Frequency distributions of BLUP W_a and CI for EG203 x MM738 DH population inoculated with strains PSS4 and R3598 in individual seasons. Frequency distributions are presented for BLUP W_a **(a)** and CI **(b)** variables in season 1 and BLUP W_a (**c)** and CI **(d)** in season 2 for PSS4 strain (Reunion assay). For strain R3598 (Cameroon assay), frequency distributions are presented for W_a **(e)** and CI **(f)** variables in season 1, and W_a **(g)** and CI **(h)** in season 2. Arrows indicate the means of susceptible parent E8 (MM738), resistant parent E4 (EG203) and their F_1 (E4×E8).

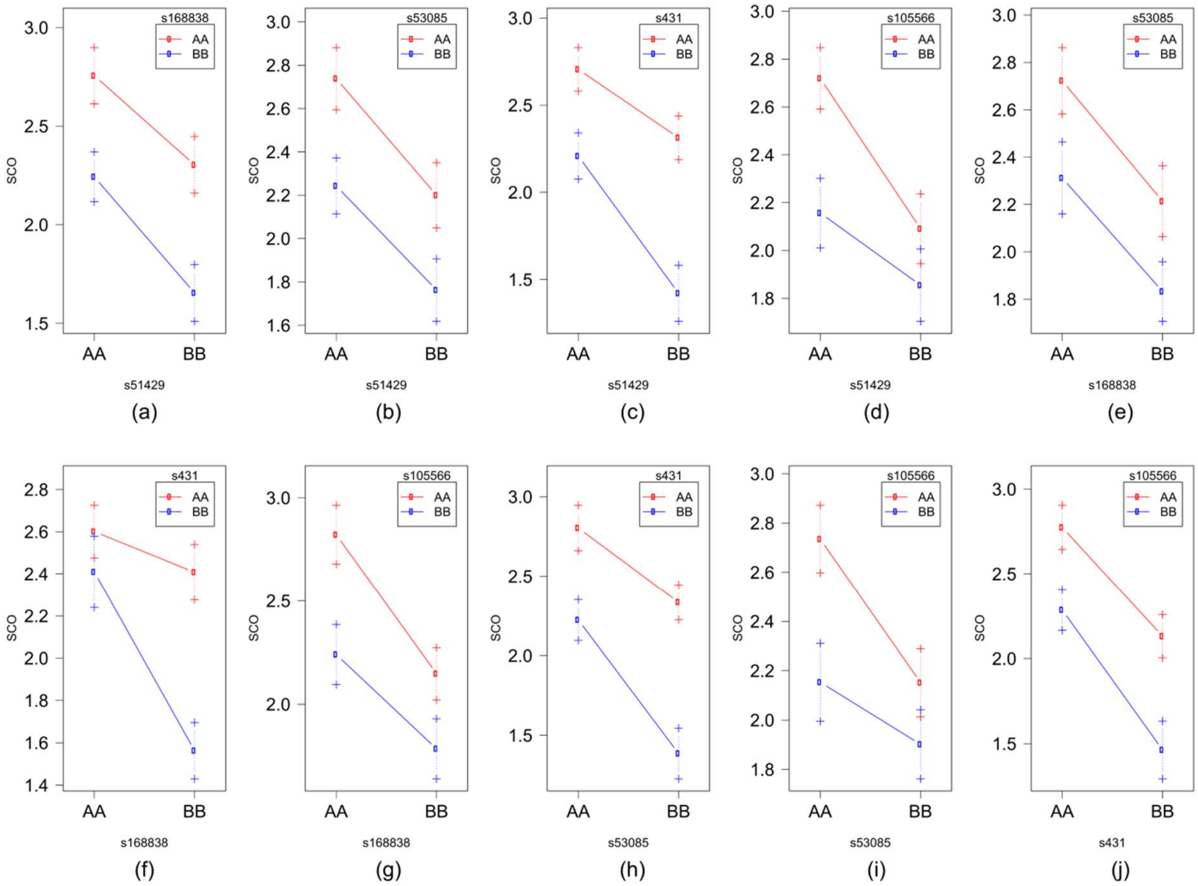

Figure S2. Effectplots for epistatic interactions between pairs of QTL detected for SCO_{max} in combined seasons of PSS4 trials.

Epistatic interactions are presented for closest markers at loci pairs: *ERPR2b/ERPR3a* **(a)**; *ERPR2b/ERPR4* **(b)**; *ERPR2b/ERPR6b* **(c)**; *ERPR2b/ERPR8* **(d)**; *ERPR3a/ERPR4* **(e)**; *ERPR3a/ERPR6b* **(f)**; *ERPR3a/ERPR8* **(g)**; *ERPR4/ERPR6b* **(h)**; *ERPR4/ERPR8* **(i)**; *ERPR6b/ERPR8* **(j).**

AA: DH lines homozygous for E8 (MM738) alleles;

BB: DH lines homozygous for E4 (EG203) alleles.

Plotted points indicate SCO_{max} means ± standard error for allelic combinations at locus 1 (X axis) and locus 2 (represented by a color code written in the rectangle at the top left corner of each sub-figure).
